# Sustainable Applications of Nanopropolis to Combat Foodborne Illnesses

**DOI:** 10.3390/molecules28196785

**Published:** 2023-09-24

**Authors:** Fernanda Wariss Figueiredo Bezerra, Jonilson de Melo e Silva, Gustavo Guadagnucci Fontanari, Johnatt Allan Rocha de Oliveira, Mahendra Rai, Renan Campos Chisté, Luiza Helena da Silva Martins

**Affiliations:** 1 Graduate Program of Food Science and Technology (PPGCTA), Institute of Technology (ITEC), Federal University of Pará (UFPA), Belém 66075-110, Brazil; jonilsonm97@gmail.com (J.d.M.e.S.); rcchiste@ufpa.br (R.C.C.); luiza.martins@ufra.edu.br (L.H.d.S.M.); 2 Instituto de Saúde e Produção Animal, Universidade Federal Rural da Amazônia, Belém 66077-530, Brazil; gustavo.fontanari@ufra.edu.br; 3 Faculdade de Nutrição, Universidade Federal do Pará, Belém 66075-110, Brazil; johnattrocha@yahoo.com.br; 4 Department of Biotechnology, SGB Amravati University, Amravati 444602, India; mahendrarai7@gmail.com

**Keywords:** food preservation, nanotechnology, propolis

## Abstract

Propolis has numerous biological properties and technological potential, but its low solubility in water makes its use quite difficult. With the advent of nanotechnology, better formulations with propolis, such as nanopropolis, can be achieved to improve its properties. Nanopropolis is a natural nanomaterial with several applications, including in the maintenance of food quality. Food safety is a global public health concern since food matrices are highly susceptible to contamination of various natures, leading to food loss and transmission of harmful foodborne illness. Due to their smaller size, propolis nanoparticles are more readily absorbed by the body and have higher antibacterial and antifungal activities than common propolis. This review aims to understand whether using propolis with nanotechnology can help preserve food and prevent foodborne illness. Nanotechnology applied to propolis formulations proved to be effective against pathogenic microorganisms of industrial interest, making it possible to solve problems of outbreaks that can occur through food.

## 1. Introduction

The term “nanotechnology” describes areas of science and engineering where phenomena that occur in nanometric dimensions (10^−9^ m) are used in systems of study in several areas. Nanotechnology can increase the capacity and quality of the industrial sector while promoting economic growth [1].

Food safety is a global public health concern since the food matrix is highly susceptible to contamination of various natures (chemical, physical, and biological), which can lead to both a decline in food quality (unacceptable sensory attributes) and transmission of harmful foodborne illness due to foods with a high load of toxins produced by microorganisms [2,3].

Propolis is a resin-like product produced by *Apis mellifera* from plant buds and exudates. This material has been reported as a promising antioxidant source containing mainly phenolic compounds, such as flavonoids, in its composition [4].

Propolis is naturally used as a defense mechanism by hives against predators. In addition to sanitizing gaps in the hive fence due to environmental conditions, propolis also acts as a thermal insulator, maintaining the humidity and temperature of the hive and preserving bees’ sanity [5,6].

Despite the numerous biological properties and potential technological applications, propolis has some limitations in its applications due to the lack of adequate formulations of this product, as well as difficulties in formulating a more stable product. For instance, its low solubility in water makes its general use quite difficult. However, with the advances in nanotechnology, there is hope in developing better formulations with improved solubility in water and aqueous systems, bioavailability, controlled release profiles, and penetration capacity [7].

The natural nanomaterial called nanopropolis has several applications, including in improving food quality. Due to their smaller size, nanoparticles of propolis have more antimicrobial effects than common propolis and are more readily absorbed by the body [7].

Therefore, for the first time, this review gathers information to determine whether using propolis in combination with nanomaterials can prevent foodborne pathogens.

For this purpose, an analysis of the literature in the Scopus, Medline, CINAHL, Cochrane Database, and Web of Science databases published in the last five years was carried out. The keywords used were nanopropolis, propolis, nanopropolis antimicrobial, and bioactive compounds, which were the descriptors used in a survey of relevant research. The main screening process was based on scientific articles, book chapters, and scientific data released in the last five years (2018 to 2023). However, older references with relevant information were added when appropriate. Each study was analyzed separately, and those related to the research theme were chosen. Importantly, publications with only abstracts that did not fall within the established publication period were not considered.

## 2. Foodborne Illness as a Public Health Challenge

Foodborne illness presents as main symptoms fever, nausea, vomiting, and diarrhea, among others, yet people may have difficulty detecting these symptoms due to their similarity with those of other illnesses, such as the flu [7].

Foodborne illnesses can affect anyone, but for some people, this risk ends up being higher than for others, which can lead to death, especially in people who are more susceptible and have low immunity. Within this niche, the most affected people may be pregnant women, young children, babies, and the elderly population [7].

Food is the main vehicle for many illnesses because it is a nutritious substrate for pathogenic microbial agents, but this depends on the chemical composition of foods, as well as storage temperatures, exposure to air, oxygen levels, and handling processes [8].

According to da Silva Martins et al. [8], among the population of pathogens that cause foodborne outbreaks, there are more than 200 types of illnesses that are known, and most of them are caused by bacteria. Foods like meat, dairy products, bakery products, eggs, and fish and crustaceans are among the main causes of illnesses when they are contaminated by microorganisms. The most common of these microorganisms are listed in Table 1 (according to the USD [9] website).

To understand the factors involved in these illnesses and their treatment, it is necessary to investigate the main aspects related to illness caused by food and its etiological agents. However, there are still several new challenges arising in investigations of emerging or re-emerging microorganisms that require detailed reporting of foodborne and waterborne outbreaks, which is crucial for illnesses control, prevention, and reporting [8].

The Centers for Disease Control and Prevention (CDC) [20] coordinates investigations into foodborne illnesses, carrying out several screenings in several states per week; among the microorganisms most commonly involved in outbreaks are the following: *Campylobacter*, *E. coli*, *Listeria monocytogenes*, and *Salmonella*. This center presents various data and tables on the most recent food outbreaks. This information is interesting, as we can observe that food outbreaks are still a problem across the globe; even in developed countries, the population can be affected by some type of food outbreak.

Natural bioactive compounds available in nature can be alternative and sustainable strategies to conventional or synthetic drugs due to their known antimicrobial activity against these types of pathogenic microorganisms. In addition, they also act against deteriorative microorganisms that decrease the shelf life of food products.

## 3. Propolis: Chemical Composition, Antioxidant, and Antimicrobial Properties

Concerning technological approaches, it is crucial to pay attention to extraction procedures, with solid–liquid extraction using ethanol and water being the most popular method to ensure the integrity of the chemical components of propolis and its biological activities as a consequence. However, classic techniques bring low yields and sometimes the use of non-green and expensive solvents that can pollute the environment (depending on the type of organic solvent). Thus, green approaches, such as supercritical extraction, have been receiving attention in recent years. Supercritical extraction with CO_2_ is characterized as being milder, more selective, and more effective at removing supercritical fluid, yet the development of the industriaNSl plants and equipment necessary for this method is expensive, and optimized conditions such as pressure, temperature, flow and volume of the solvent, type, and concentration of co-solvents must be considering carefully to recover any target compound [21].

In addition to being used as a natural medicine for several illnesses, propolis has wide-ranging technological applications, and it can also be used as a preservative for food and meat, as its action against pathogenic microorganisms has already been proven and is widely recognized nowadays [22].

The antioxidant and biological properties of propolis are positively linked to its chemical and phytochemical composition. Factors such as collection season, the type of plants that bees visit, the location of hives, and post-harvest processing are extremely relevant to its quality [23].

Pobiega et al. [24] reported promising antimicrobial activity in propolis that can minimize or eliminate foodborne pathogenic microorganisms. Flavonoids, together with phenolic acids and esters, phenolic aldehydes, and ketones, are considered the most important antimicrobial compounds of propolis. Regarding other compounds, we have volatile oils and aromatic acids, waxes, resins, balsam, and pollen, which is a rich source of essential elements such as magnesium, nickel, calcium, iron, and zinc. The mechanism of antibacterial activity is considered complex and can be attributed to the synergism between flavonoids, hydroxy acids, and sesquiterpenes. 

In addition, propolis possibly has antibacterial action, increasing the body’s immunity or acting against the microorganism of interest. The phenolic compounds of propolis act effectively against Gram-positive bacteria due to the membrane structure of these microorganisms [25].

The composition of propolis is better explained in the section “Main chemical agents involved in the bioactivity of propolis”.

It is important to consider the factors that interfere in the chemical composition of propolis since it is not a stable product with a predictable chemical composition but a natural product with high biological and commercial value. Concerning its biological potential, phenolic acids and flavonoids are the most-studied classes of bioactive compounds that confer a high antioxidant capacity on propolis [26].

According to Sahu et al. [26], propolis is a natural antioxidant acting against free radicals that can also cause illnesses, such as weakening of the body systems and aging, as well as more serious disorders, such as arthritis, arteriosclerosis, and cataracts, among others. These authors found that nanopropolis is an excellent drug that can be used in the pharmaceutical and food industries.

The high concentrations of pinocembrin present in Chilean propolis demonstrated antibacterial action, with inhibition against the genes GtfB, GtfC, and GtfD, which are related to biofilm formation in strains of *Streptococcus mutans* present in children’s caries. The mechanisms of the antimicrobial action of propolis extracts, according to Fitria et al. [27], cause physical changes in the cell membrane of Gram-positive and Gram-negative bacteria, causing deformation and leakage of cellular components.

## 4. Main Chemical Agents Involved in the Bioactivity of Propolis

The polyphenols present in propolis are responsible for its biological activity. As already mentioned, this study aims to address them in more detail. We must bear in mind that these compounds are categorized into different classes according to the main basis of their chemical structure. The most common ones are isoflavones, flavonols, anthocyanidins, tanshinones, and prenylated flavonoids [28].

Flavonoids are represented by the classes of plant phytoestrogens derived from phenylpropanoids; among them, more than 10,000 different compounds with biological activity have already been studied, which are also divided into six subclasses: flavonols, flavones, flavanones, flavanols, anthocyanins, and isoflavones [28].

In plants, these compounds are related to the protection of plants against ultraviolet irradiation and the defense against different pathogens and insect pests, existing predominantly as a conjugated form of biologically inactive β-glucoside, and flavonoids and isoflavones have the highest degree of bioavailability among all the flavonoid subclasses [28].

According to the study by Fitria et al. [27], the main compounds present in propolis are shown in Table 2.

## 5. Methods of Synthesis of Nanopropolis and Its Antimicrobial Effects 

There are several methods used for the elaboration of nanopropolis, and the advantages of propolis in nanopropolis have been observed to be accentuated due to its smaller size, which increases its antioxidant and antimicrobial actions. Below we have listed some methods for the elaboration of nanopropolis. 

Table 3 provides a summary of the antibacterial activity, techniques for generating nanopropolis, and evaluation of its antimicrobial activity. 

**Figure 1:**
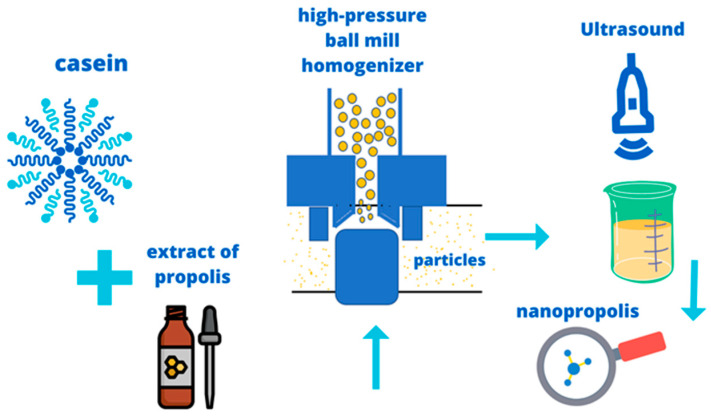
Scheme of production of nanopropolis according to Barsola and Kumari [45].

Figure 1 shows a diagram of the preparation of nanopropolis using a high-pressure homogenizer followed by ultrasound, a technique that has shown excellent results.

Selvaraju et al. [22] prepared particles of nanopropolis with silver from an extract of alcohol + propolis and distilled water in a 7:3 ratio using an ambient temperature of 25 °C under agitation for a period of 7 to 10 days in the dark, which was filtered. The filtrate was then evaporated using a rotary vacuum evaporator. This material was subjected to filtration and evaporated using a rotary evaporator. This extract was mixed with an amount of silver nitrate (AgNO_3_) and ultrapure water. Initially, the solution showed a light-brown color, and with the addition of AgNO_3_, it turned dark brown (which is indicative of the formation of nanoparticles). This material was allowed to rest, an important process since it guarantees the oxidation of silver nitrate into silver ions. The synthesized solution went through the centrifugation process, and the sediment was collected and then subjected to the lyophilization process, producing silver and propolis nanoparticles.

Seibert et al. [51], in their study on propolis extracts, showed good antimicrobial activity against the following microorganisms: *L. monocytogenes*, *S. aureus*, *S. saprophyticus*, and *E. faecalis*. It should be considered that propolis produced via nanoemulsion, due to its smaller particle size, can act more effectively against these microorganisms than propolis in the form of an extract. Therefore, even if using it as an extract is an intriguing strategy, its nanoparticle form is preferable.

Selvaraju et al. [22] applied silver nanopropolis in a non-food matrix, although this application was not effective. The authors further observed the activity of these nanoparticles against microorganisms of interest in food, such as Staphylococcus aureus. It is well known that such bacteria can cause staphylococcal poisoning. Foods such as milk, cream, pies filled with cream, potato salads, tuna, chicken, and cooked ham are among the most common foods contaminated by *S. aureus* and causing illness in humans. The main symptoms are nausea, vomiting, abdominal cramps, diarrhea, and sweating. In the research by Selvaraju et al. [22], it was observed that there was a synergistic effect between silver nanoparticles and propolis against S. aureus, and an increase in antibacterial activity.

Moreover, these materials alone are not enough to protect food from possible chemical and biological changes, requiring an active ingredient with components that can act together to protect the food.

**Figure 2:**
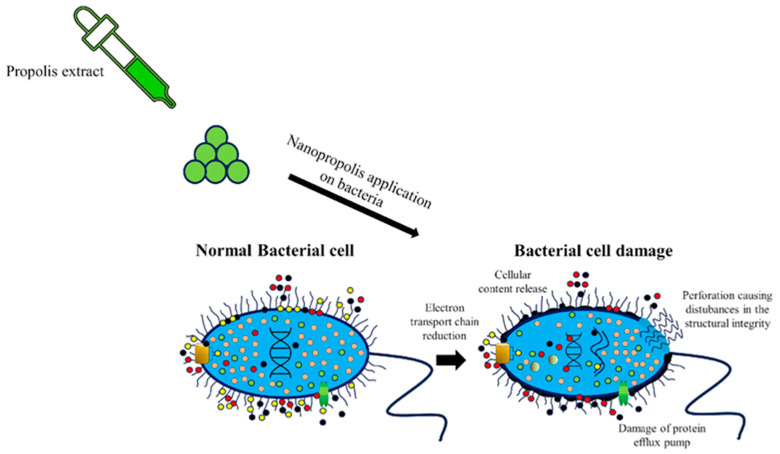
Effects of antibacterial activity on the bacterium cell.

In a study by Hasan et al. [52], nanopropolis was 206, 212, 227, and 230% more efficient against *S. aureus*, *E. coli*, *Salmonella* sp., and *Bacillus subtilis* than common propolis. The antibacterial activity of nanopropolis was evaluated in comparison to the antibiotic ampicillin, which was used as a positive control in the same investigation. All the mentioned microorganisms are known to be involved in illness transmitted by contaminated food (Figure 2).

There are some hypotheses about the antimicrobial action of propolis, such as that proposed by Sabir [53], who reports that some constituents of propolis (phenolic compounds and flavonoids) may possibly prevent a bacterial enzyme called RNA polymerase from binding to the DNA of the bacteria, which prevents its replication by having the enzyme-restrictive endonuclease. Another hypothesis is that there may also be a reduction in the electron transport chain, causing perforation and thus disturbances in the structural integrity of the cell. As propolis is not very soluble, nanopropolis could be more effective when we make a comparison between the two, since nanopropolis can more easily penetrate the outer membrane of bacteria and its antimicrobial agents would work more effectively against their intended targets [7].

Mei et al. [4] conducted a study on the ability of an ethanolic extract of propolis and phosphatidylcholine generated via nano-microencapsulation to protect tea seed oil. The authors studied the antioxidant activity of nano-microcapsules, and the results showed that the nano-microcapsules had good characteristics in terms of nanostructure, morphology, and interface, and nano-microencapsulation considerably improved the antioxidant activity of the microcapsules, showing its potential to be applied for the protection of edible oil in the food industry.

Júnior et al. [54], in a study using green propolis extract and silica nanoparticles (SiO_2_) in the structure of films based on sodium alginate, evaluated their physical and antioxidant properties compared with a control sample. The incorporation of propolis provided an excellent UV light-blocking effect and intense DPPH radical scavenging activity for all samples; thus, propolis with (SiO_2_) nanoparticles has been proven to have potential for future applications in active food packaging.

Shahabi et al. [55] investigated the effect of halloysite nanotubes on the physicochemical characteristics of propolis-activated soy protein isolate/basil gum film. In addition to achieving excellent results in film analyses, the authors observed a significant increase in the antimicrobial and antioxidant potential of the samples (in tests of DPPH radical scavenging activity) when adding propolis. In conclusion, the films produced showed acceptable efficiency for use in a food packaging system.

Soleimanifard et al. [56] studied a bioactive composition/mixture with potent pharmacological efficacy using propolis extract. In this study, propolis was extracted and trapped in a sodium caseinate–maltodextrin nano-complex. The results proved helpful in formulating nanocomplexes suitable for propolis extract applications in food and pharmaceutical products.

Madani et al. [57] reported the antimicrobial efficacy of propolis and propolis nanoparticles (NPs) against *E. faecalis* biofilm in vitro. The results indicated that NPs, in a concentration ten times lower, were more effective against *E. faecalis* (PTCC 1778) than common propolis.

## 6. Advantages and Disadvantages of Using Nanopropolis in the Food Industry

Propolis has great potential to be used as a food preservative due to its antimicrobial and antioxidant properties and because it is natural and recognized as a safe substance that can be applied in foods such as meat, beverages, dairy products, fruit juices, and eggs, among others. But there is a limiting factor in the use of propolis as a food preservative due to its flavor (it has high astringency and bitterness), which affects consumer acceptability, which occurs due to the high content of polyphenols present [28].

Seibert et al. [51] reports that a way to solve this problem would be the encapsulation of propolis in nanoemulsions, which would minimize the impact of negative sensory properties for consumers. A nanoemulsion is defined as a colloidal dispersion with droplet sizes ranging from 20 to 200 nm, formed through droplets of a liquid dispersed in another immiscible liquid which are stabilized by surfactants.

**Figure 3:**
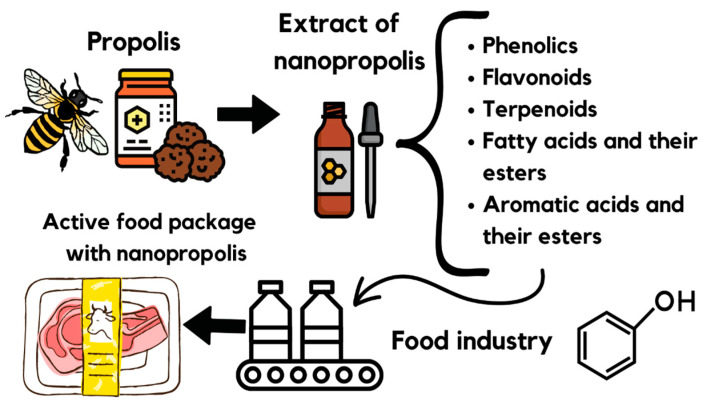
Application of nanopropolis in food packaging materials based on biopolymers.

But we realized the best application for nanopropolis in food would be to couple it with packaging materials based on biopolymers, as these are biodegradable, biocompatible, non-toxic, and have a broad spectrum of activity (Figure 3). Biopolymers may comprise proteins and polysaccharides, which are promising materials [4].

By altering the physical characteristics of packaging materials and adding bioactive chemicals, nanotechnology enables the creation of intelligent packaging for meals with certain desirable attributes.

## 7. Nano-Delivery Systems (NDSs) in Food

Materials in the nanoscale range are used in nano-delivery systems (NDSs), a relatively new but quickly evolving field of research, to transport bioactive substances or agents to targeted areas in a regulated way [58].

Regarding nano-delivery systems (NDSs), the chemical properties depend on the type of delivery system. NDSs are composed of materials already approved for food, such compounds including lipids, proteins, saccharide polymers, lactic acids, and combinations thereof [59].

We summarize the interesting nanocarriers used for delivery systems and their particularities in Table 4 [59].

## 8. Safety in the Use of Nanopropolis in Food

The biggest concern of scholars in relation to the safety of using nanoparticles in food is the phenomenon of their migration in food. Elements such as nanoragila nanosilver, titanium nanoparticles, and other elements can migrate to food; however, the conclusions from different studies still conflict, perhaps due to failures in the planning of the experiments carried out, which ended up generating an alert from government agencies. Since the use of nanotechnology is accelerating, this concern is important [63].

The phenomenon of migration is a concern as it could compromise the health of the consumer, for example, due to metals that can normally migrate into food. There are three stages in the migration process, namely (1) diffusion, (2) dissolution, and (3) dispersion of the nanoparticle; thus, understanding this process is of great importance for determining the health risks when such compounds encounter food products [63].

However, factors such as the chemical composition of the food, composition of the packaging material, mechanical and storage conditions, and handling are important in evaluating the possible interactions of nanoparticles with the food [59].

Factors such as size, chemical composition, crystallinity, particle characteristics, and surface functionality are important in determining the toxicity of nanoparticles. Many nanoparticles differ in their toxicological properties due to the method of synthesis used and the compounds present in the environment into which they are inserted [59].

One of the most promising arguments for the use of nanopropolis is that its base is a natural compound, and therefore there is no chance of toxicity to humans as it is edible and generally recognized as safe (GRAS). The use of natural compounds has been explored due to their numerous benefits and non-toxic effects on humans. However, this is not generalizable; it should be kept in mind that sensitive people may be affected by natural components [64].

## 9. Conclusions and Future Perspectives

Propolis is known for its biological properties and benefits for human health. Nanotechnology combined with propolis has proved to be effective in food preservation in the consulted studies, presenting bioactivity against microorganisms that cause foodborne illness, and thus being a promising compound in the food industry.

For nanopropolis to be used in the food industry, nanotechnological interventions are needed to enhance the performance of this promising compound’s key properties, including increasing its aqueous solubility, bioavailability, controlled release profile, and penetration capacity. When there is a mismatch between the dose and available concentration of a medicinal substance and its efficacy, the application of nanotechnology is brought into question.

In this way, the use of nanopropolis as an emerging technology has brought advantages, such as its use in packaging as a controlled release system for active ingredients, nutrients, antioxidant compounds, and/or antimicrobial agents in pharmaceuticals, cosmetics, fine chemicals, and food products, adding greater value due to its potential for improving mechanical, thermal, physicochemical, biological, and biodegradability characteristics, as well as its ability to act as a freshness indicator and extend the shelf life of products.

## Figures and Tables

**Table 1:** Main microorganisms involved in food outbreaks, foods where they occur, and occurrences of outbreaks.

Type of Microorganism	Type of Food	Symptoms	Current Outbreaks	Reference
*Campylobacter jejuni*	Shellfish, milk, chicken, and meat	Gastrointestinal disorders, diarrheal illness, and prosthetic joint infection (rare)	China	Zulqarnain et al. 2022 [10]
*Clostridium botulinum*	Cheese products, canned vegetables, canned meats	Neurotoxins, disease (neurological symptoms)		Chaidoutis et al. 2022 [11]
*Clostridium perfringens*	Animals’ and humans’ intestinal tract, food such as raw meats, dehydrated soups, and sauces, raw vegetables, and spices	Diarrheal illness, necrotizing enteritis	England	Dolan et al. 2016 [12]
*Cryptosporidium*	Water, fruit, vegetable, and shellfish contamination, calves and cheese curds	Acute diarrhea, vomiting, nausea, and abdominal pain	France	Costa et al. 2022 [13]
*Escherichia coli* O157:H7	Cattle and other ruminants and contaminated food or water	Severe bloody diarrhea and hemolytic uremic syndrome	Middle East/North Africa	Cowley et al. 2016 [14]
*Listeria monocytogenes*	Ready-to-eat meat and poultry products	Mild gastroenteritis and invasive listeriosis	USA, European Union, Australia, New Zealand	Zhang et al. 2021 [15]
*Staphylococcus aureus*	Dairy products (milk, cheese, and cream), meat, and fish	Sudden vomiting, diarrhea, nausea, malaise, abdominal cramps, pain, and prostration	Vietnam	Le et al. 2021 [16]
*Vibrio* spp. *Vibrio parahaemolyticus* and *Vibrio vulnificus*	Water and filter-fed shellfish, especially oysters	Cholera, severe extraintestinal infections, including necrotizing fasciitis and septicemia, and deaths	Japan and USA	Brumfield et al. 2023 [17]
*Salmonella enterica serovars*	Meat, poultry, fruits, and vegetables	Gastroenteritis, bacteremia, extraintestinal infection, infectious osteomyelitis, and discitis	USA	Reddy, Sulaiman and Askar 2023 [18]
*Norovirus*	Packaged delicatessen meat	Gastroenteritis, diarrhea or vomiting	USA	Malek et al. 2009 [19]

**Table 2:** Phenolic compounds isolated from propolis and reported biological properties.

Phenolic Compound	Type	Chemical Structure	Biological Properties	Reference
Pinocembrin or 5,7-dihydroxyflavanone	Flavonoid/flavanone	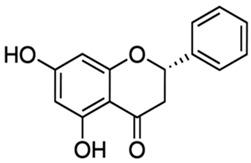	Antioxidant, anti- inflammatory, and antibacterial activities	Chen et al. 2022 [29]
Chrysin or 5,7-dihydroxyflavone	Flavonoid/flavone	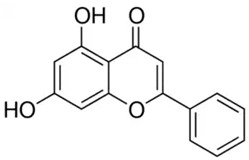	Antioxidant, anti-inflammatory, anticancer, and antiviral activities	Mani and Natesan 2018 [30]
Apigenin or 4′,5,7-trihydroxyflavone	Flavonoid/flavone	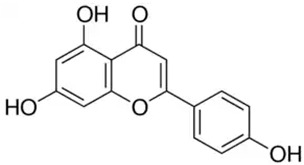	Anti-inflammatory and antioxidant functions	Salehi et al. 2019 [31]
Caffeic acid phenethyl ester in (CA)	Phenolic acid/hydroxycinnamic acid	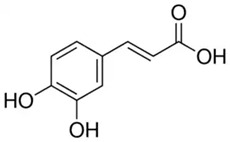	Antioxidant and anticancer activities	Pagnan et al. 2022 [32]
Vanillin or 4-hydroxy-3-methoxybenzaldehyde or vanillic aldehyde	Phenolic acid/phenolic aldehyde	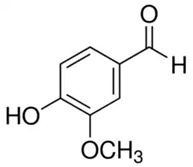	Neuroprotective, antioxidant, and anti-inflammatory activities	Iannuzzi et al. 2023 [33]
Syringic acid (SA) or 3,5-Dimethoxy-4-hydroxybenzoic acid	Phenolic acid/benzoic acid derivative phenolic	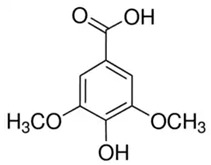	Antioxidant, anti-inflammatory, and neuroprotective activities	Ogut et al. 2022 [34]
Pinobanksin or (2R,3R)-3,5,7-Trihydroxy-2-phenyl-2,3-dihydro-4H-chromen-4-one	Flavonoid/flavanone	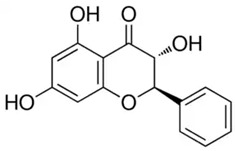	Antimicrobial, antioxidant, and anti-inflammatory activities	Widelski et al. 2023 [35]
Cinnamic acid or (Z)-cinnamate or 3-phenyl-acrylate	Phenolic acid hydroxycinnamic acid	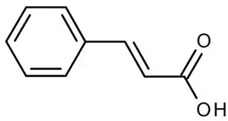	Anticancer, antioxidant, and anti-inflammatory activities	Feng et al. 2022 [36]
Rutin or 3,3′,4′,5,7-Pentahydroxyflavone 3-rutinoside	Flavonoid/flavonol glycoside	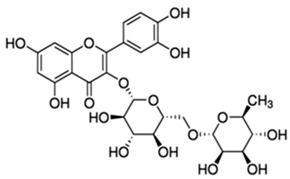	Antioxidant and anti-inflammatory activities	Muvhulawa et al. 2022 [37]
Artepillin C (ARC) or (2E)-3-[4-Hydroxy-3,5-bis(3-methyl-2-buten-1-yl)phenyl]-2-propenoic acid	Phenolic acid/prenylated derivative of p-coumaric acid	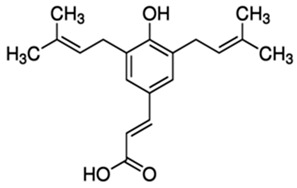	Antioxidant, antimicrobial, anti-inflammatory, antidiabetic, neuroprotective, and gastroprotective activities	Shahinozzam et al. 2020 [38]
Protocatechuic acid or 3,4-Dihydroxybenzoic acid or protocatechuic acid	Phenolic acid/hydroxybenzoic acid	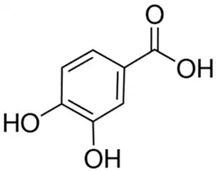	Antioxidant activity, antibacterial activity, anticancer activity, antiulcer activity, antidiabetic activity, antiaging activity, antifibrotic activity, antiviral activity, anti-inflammatory activity, analgesic activity, antiatherosclerotic activity, cardiac activity, and hepatoprotective activity	Kakkar and Bais 2014 [39]
Chlorogenic acid (CGA) or 1,4,5-Trihydroxycyclohexanecarboxylic acid 3-(3,4-dihydroxycinnamate), 3-(3,4-Dihydroxycinnamoyl) quinic acid	Phenolic acid/hydroxycinnamic acid	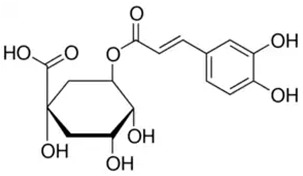	Antioxidant, antibacterial, hepatoprotective, cardioprotective, anti-inflammatory, antipyretic, neuroprotective, antiobesity, antiviral, antimicrobial, and antihypertension activities; free radical scavenger; and a central nervous system stimulator	Naveed et al. 2018 [40]
Isorhamnetin or 3′-Methoxy-3,4′,5,7-tetrahydroxyflavone	Flavonoid/flavanone	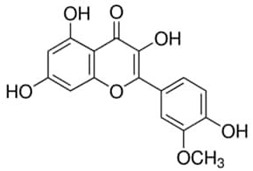	Cardiovascular and cerebrovascular protection; antitumor, anti-inflammatory, and antioxidation activities; organ protection; and prevention of obesity	Gong et al. 2020 [41]
Liquiritigenin or 7,4′-Dihydroxyflavanone	Flavonoid/flavanone	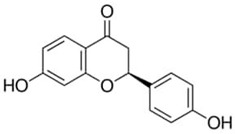	Antidiabetic, anticancer, hepatoprotective, antibacterial, and anti-inflammatory activities; radical scavenging; neuroprotection against stroke; and memory-enhancing activities	Ramalingam et al. 2018 [42]
Formononetin or 7-Hydroxy-3-(4-methoxyphenyl)-4H-1-benzopyran-4-one	Flavonoid/O-methylated isoflavone	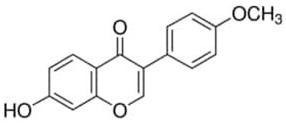	Anticancer, antibacterial, anti-inflammatory, and antioxidant activities	Tay et al. 2019 [43]
Biochanin A or 4′-Methylgenistein, 5,7-Dihydroxy-4′-methoxyisoflavone	Flavonoid/O-methylated isoflavone	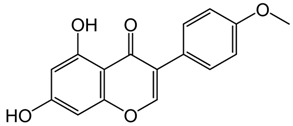	Anticancer, antibacterial, anti-inflammatory, antioxidant, and neuroprotective activities	Morissette et al. 2018 [28]
Myricetin or 3,3′,4′,5,5′,7-Hexahydroxyflavone	Flavonoid/flavonol	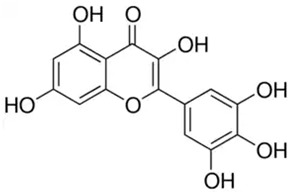	Antidiabetic, anticancer, hepatoprotective, antibacterial, and anti-inflammatory activities	Eddouks et al. 2014 [44]

**Table 3:** Nanopropolis elaboration techniques and antimicrobial action.

Obtaining the Extract	Method for Obtaining Nanopropolis	Nanoencapsulation Agent	Antimicrobial Action	Reference
Propolis with ethanol extract	Using the high-speed and high-pressure homogenization method, after homogenization, this material was evaporated to remove solvents, thus obtaining the propolis or nanopropolis nanoparticles.	-	not determined in this research	Tatli et al. 2018 [7]
-	A high-pressure ball mill homogenizer was used to produce nanosized particles, which were then subjected to sonication and separated using an ultrafiltration system, synthesizing the nanoparticles.	Casein micelles	not determined in this research	Barsola and Kumari 2022 [45]
Propolis with ethanol extract	Agitation via magnetic stirring and heating at 60 °C until complete solubilization were carried out to create nanopropolis suspensions; this study aimed at creating nanoparticles while altering the quantities of propolis extract to optimize the preparation conditions.	Soy lecithin	not determined in this research	Pinheiro Machado et al. 2019 [46]
Propolis with ethanol extract	The extracting solution was subjected to magnetic stirring for 7 days at room temperature; then, it was filtered with Wattman 4 paper to separate the impurities. For, to this solution, water in a 1:10 ratio was added for the purification of propolis. The suspension was submitted to an ultrasonic bath for 30 min until obtaining colloidal nanoprolis, with a pH adjusted to neutral. In the final step, the authors converted the colloidal nanopropolis into powder through the lyophilization process, thus obtaining nanopropolis particles.	Oil and surfactants	Not determined in this research	Zaleh et al. 2022 [47]
Propolis with ethanol extract	Obtained via the milling media method	Not mentioned	*Staphylococcus aureus* and *Candida albicans*	Afrouzan et al. 2012 [48]
Propolis with ethanol extract	High-pressure ball mill homogenizer	Casein micelle	*Escherichia coli*, *Bacillus subtilis*, and *Staphylococcus aureus*	Hamdi et al. 2019 [49]
Propolis with ethanol extract	High-speed homogenization technique and solvent evaporation	Casein micelle	*B. subtilis, for S. aureus* and *E. coli*	Prasetyo 2019 [50]

**Table 4:** Nanocarriers used for delivery systems and their particularities.

Material	Functionality	Advantages	Reference
Protein-Based Systems	Food proteins are frequently employed in manufactured foods since they are GRAS and have a high nutritional value. The most practical and often-utilized matrix in food applications is protein hydrogel. It is possible to create novel protein vehicles with increased delivery capabilities by shrinking the matrix size from micrometers to nanometers.	Protein-based NDSs can be made in two ways that are both relatively simple to prepare: the “top-down” approach, where structures are created by disassembling bulk materials, and the “bottom up” approach, where structures are created from molecules that are capable of self-assembly. Because they may form complexes with polysaccharides, lipids, or other biopolymers and a range of nutrients can be added, protein-based NDSs are particularly intriguing.	Augustin 2003 [60]
Polysaccharide- and Poly(lactic) Acid-Based Systems	Glycosidic linkages hold the monosaccharides (carbohydrates) in polymers called polysaccharides. These naturally occurring polymers, which can be found in pectin, guar gum, and insulin from plants, chitosan and chondroitin sulfate from animals, alginates from algae, and dextran from microbes, are often quite big and frequently branched.	To provide controlled release carriers for medications and proteins, homo- and copolymers made of poly (lactic acid) and poly (glycolic acid) are widely used because of their biodegradability and biocompatibility. These aliphatic polyester polymers are broken down by bulk ester bond hydrolysis.	Chayed and Winnik 2007 [61]
Lipid-Based Systems	These systems provide a few benefits over conventional encapsulation techniques, including the potential for industrial-scale production using natural components, targetability, and the capacity to entrap substances with various solubilities.	The structure of liposomes can include additional molecules like proteins or polymers in addition to one or more lipid and/or phospholipid bilayers. The ability of nanoliposomes to concurrently absorb and release two compounds with different solubilities is a key benefit. This indicates that these systems can tolerate both lipid-soluble and water-soluble molecules.	Taylor et al. 2005 [62]

## Data Availability

The research outlined in the article did not include any data.
